# Dried Blood Spot-Based Quantification Indicates Correlation Between lncRNA GAS5, Diabetes, Age, and War Eras in US Veterans

**DOI:** 10.3390/biology15171488

**Published:** 2026-09-02

**Authors:** Meredith Krause-Hauch, Rekha S. Patel, Bangmei Wang, Lauren Deland, Stephen Mastorides, Rahul Mhaskar, Niketa A. Patel

**Affiliations:** 1 James A. Haley Veterans’ Hospital, 13000 Bruce B. Downs Blvd., Tampa, FL 33612, USA; meredith.krause-hauch@va.gov (M.K.-H.); rekha.patel1@va.gov (R.S.P.); bangmei.wang@va.gov (B.W.); lauren.deland@va.gov (L.D.); stephen.mastorides@va.gov (S.M.); rahul.mhaskar@va.gov (R.M.); 2 Department of Internal Medicine and Medical Education, Morsani College of Medicine, University of South Florida, 4202 E. Fowler Ave., Tampa, FL 33620, USA; 3 Department of Molecular Medicine, Morsani College of Medicine, University of South Florida, 4202 E. Fowler Ave., Tampa, FL 33620, USA

**Keywords:** dried blood spots, Vietnam war veterans, type 2 diabetes, lncRNAs, GAS5, Agent Orange, TCDD, biomarker

## Abstract

US Veterans are disproportionately affected by type 2 diabetes (T2D) compared with civilians. Previously, we demonstrated that serum GAS5 levels are lower in Veterans with T2D compared with non-diabetics and established GAS5 as a genetic biomarker for T2D, validated by others. Dried blood spots (DBS) are advantageous because collection is minimally invasive, and shipment can be at room temperature. Measurement of lncRNAs in DBS is not yet reported and hence we sought to measure lncRNA GAS5 levels in DBS collected from Veteran participants. Results demonstrate that older age was independently associated with lower GAS5 levels among Veterans. Comparing Veterans across war eras, in this cohort, Vietnam War (VW) Veterans had higher incidences of diabetes compared to Gulf War (GW) or Other (non-VW/GW) wars. VW Veterans with A1c values greater than 6.5 had lower GAS5 levels, independent of BMI. Among the VW Veterans, a very small number of Veterans with self-reported Agent Orange (AO) exposure had lower GAS5 levels. Based on this observation, a mouse model with acute TCDD exposure was evaluated for Gas5 levels. The results demonstrate a significant negative correlation between TCDD dose and Gas5 levels measured in blood and DBS in the mouse model. Overall, the study demonstrates that lncRNA GAS5 measured in DBS and blood may be used in biomarker studies in diabetic patients. These results support the use of DBS in longitudinal studies of chronic diseases in active-duty service members or Veterans with toxic exposures during military service.

## Simple Summary

US Veterans have significantly higher rates of type 2 diabetes (T2D) prevalence compared with civilians. Previously we established that low levels of lncRNA GAS5 served as a blood biomarker for T2D, which is also validated by other groups. Dried blood spots (DBS) are a minimally invasive method for sample collection. In this study, we evaluated the feasibility of measuring lncRNA GAS5 from DBS, which has not yet been reported. Results show that in this cohort, older Veterans had lower GAS5 levels. Veterans of the Vietnam War (VW) had significantly lower GAS5 levels compared to Gulf War (GW) or other war era Veterans. This lower GAS5 was correlated with increased prevalence of T2D in VW Veterans compared to other Veterans. VW Veterans were exposed to the herbicide Agent Orange, which was contaminated with the toxic dioxin TCDD, previously shown to promote T2D. Based on the observation of low GAS5 levels, a mouse model with acute TCDD exposure was evaluated for Gas5 levels. Blood Gas5 levels were negatively correlated with TCDD doses in the acute mouse model. Overall, the study demonstrates the use of DBS to measure lncRNAs. Thus, DBS collection can be incorporated in the field for active service members and for Veterans in remote locations to facilitate detection of genetic biomarkers of chronic diseases.

## 1. Introduction

Approximately 1 in 4 Veterans have been diagnosed with type 2 diabetes (T2D) [1], a prevalence significantly higher than that in civilians (1 in 10) [2]. T2D is a chronic disease characterized by elevated blood glucose and insulin resistance, often associated with chronic co-morbidities such as heart disease, kidney disease, retinopathy, nerve damage, and cognitive impairment [3]. Obesity and older age are risk factors for T2D. While obesity promotes T2D, it is known that lean individuals with a normal BMI (18.5–24.9 kg/m^2^) may also develop T2D. Genetic factors account for 10–15%, of the risk for T2D development; however, other factors such as environment or lifestyle also influence T2D development [4].

Long noncoding RNAs (lncRNAs) regulate essential biological networks, and function in epigenetic regulation, transcription, splicing and translation. Aberrant expression patterns of lncRNAs are linked to human diseases. Previously [5], we undertook an unbiased screen to identify lncRNAs in serum that correlated to T2D in patients. We evaluated blood samples from Veteran participants at James A. Haley Veterans’ Hospital (JAHVH; Tampa, FL, USA). Through transcriptomics screening of lncRNAs, we discovered that serum levels of lncRNA growth-arrest specific transcript 5 (GAS5/Gas5 (mu)/SNHG2) were lower in participants with T2D compared with non-diabetic participants. Our results showed that individuals with an absolute GAS5 level below 15 ng/μL had almost twelve times higher odds of having diabetes. This study established lncRNA GAS5 as a genetic biomarker for T2D, which is now validated by other groups [6,7,8,9]. Subsequently, we and others demonstrated that GAS5 is causal to T2D, showing that GAS5 regulates insulin receptor signaling and glucose uptake [10]. Xu et al. demonstrated that GAS5 levels were low in models of diabetic nephropathy [11]. GAS5 polymorphism is associated with coronary heart disease [12] and diabetic retinopathy [13]. GAS5 has also been shown to be a part of a network associated with carotid atherosclerotic plaques in T2D [14]. Parvar et al. showed that low GAS5 levels in patients with T2D were elevated following metformin treatment [15]. GAS5 is now established as a pivotal lncRNA for T2D [16,17,18].

The current study aims to evaluate GAS5 levels from dried blood spots (DBS) in U.S. Veterans. DBS testing has been widely used in the detection of proteins, metabolites and viral infections [19]. Performing blood tests using DBS rather than plasma or serum offers several advantages: DBS samples require far less blood, and collection is minimally invasive, low-cost and can be shipped at ambient temperatures. These advantages make DBS ideal for testing samples in the field or in populations that are difficult to reach. The quantification of lncRNAs in DBS for evaluating the prevalence of chronic disease has not been previously described in the literature. In this study, we evaluated GAS5 levels in DBS samples collected from participating US Veterans and analyzed the results in the context of war era. We further evaluated the effect of acute TCDD exposure on Gas5 levels and its correlation with blood glucose levels, using a direct blood test in a mouse model.

## 2. Materials and Methods

### 2.1. Study Population

The research biospecimen repository (RBR) at the James A. Haley Veterans’ Hospital, Tampa, FL, collected blood spots under informed consent (IRB #Pro00012108) from Veteran participants between 30 April 2014 and 3 August 2014. The RBR provided the PI with the de-identified, coded samples along with demographic information such as age, sex, and racial background. Additional research parameters, such as Veteran War era (i.e., Vietnam War, Gulf War, or Other), diabetes diagnosis status, A1c value at time of DBS collection, and self-reported AO exposure status, were also included. Diabetic status was determined by searching the patient chart data for type 2 diabetes ICD-9 codes. These data were provided to the lab along with the samples. Veterans of any age (>18), race, or sex were included in this study. Veterans were excluded if they had been diagnosed with any cancer as the disease and chemotherapy treatments may alter GAS5 levels. A total of 186 samples met these criteria.

### 2.2. Dried Blood Spot (DBS) Sample Preparation and RNA Isolation

Blood spots were collected in GenPlates™ (manufacturer GenTegra^®^, Pleasanton, PA, USA, distributor ITSI-Biosciences, Johnstown, PA, USA) from 186 participating Veterans by the RBR. GenPlates™ (384-wells in 6 region format; each containing 40 wells/elements of 1 mm; 1 region per Veteran participant), are a high density, proprietary format for transporting biosamples at ambient temperatures, in the dry-state. Each plate is foil sealed at the bottom. A 10 μL blood sample was placed in the center of each well, and GenPlates were dried with GenTegra Fast dyer. The plates were top-sealed with a clear adhesive storage plate seal. Foil and plastic seals allow multi-aliquot storage using a single container while maintaining single-aliquot use. The plates were stored at −80 °C in airtight, sealed containers. RNA was extracted from DBS after 8–10 years using our optimized protocol. Five 1 mm punches were collected per extraction into in 1.5 mL in RNase-free microfuge tubes, with extractions repeated 3 times independently for a total of 15 punches per sample. A mixture of 90 μL nuclease-free water and 10 μL of RNase-Out recombinant ribonuclease inhibitor (Invitrogen, Waltham, MA, USA, catalog # 10777019) was added to the punches and agitated for 10 min. The supernatant was transferred to a fresh RNase-free microfuge tube and 400 μL of Trizol LS (ThermoFisher, Waltham, MA, USA, catalog # 10296028) was added. Trizol LS was used as it is specially formulated and shown to have excellent results for isolating RNA from liquid (supernatant) samples. RNA was extracted per manufacturer’s instructions. Genomic DNA digestion was performed using RNA Clean and Concentrator kit (Zymo Research, Irvine, CA, USA, catalog # R1014): 5 μL of DNase I (1 U/μL) and 5 μL of DNA digestion buffer were added to 40 μL RNA sample, mixed by gentle inversion, and incubated at room temperature (22 °C) for 15 min. The samples were then processed for RNA clean-up and concentration using the Zymo-Spin IC columns, included in the previously mentioned kit, following manufacturer’s instructions. The eluted RNA concentration was measured via TapeStation and Nanodrop. The samples ranged in concentration from 200–460 ng/μL, RIN 7.3–8.1, and 260/280 ratio of 1.8–2.0. The RNA was used in RT-qPCR to measure GAS5 levels.

### 2.3. Quantitative Real-Time Polymerase Chain Reaction (RT-qPCR) of Human DBS Samples

500 ng RNA was used to synthesize cDNA via BioRad iScript kit (Bio rad, Hercules, CA, USA, catalog #1708841). RT-qPCR was performed in triplicate using 2X Maxima SYBR Green Mastermix with ROX (Thermo Scientific, Waltham, MA, USA, catalog #A25742) on an Applied Biosystem’s Viia7 RT-qPCR machine (Applied Biosystems, Carlsbad, CA, USA) using 1 μL cDNA per reaction. GAPDH was used as the endogenous control for human samples. Primer sequences: Human GAS5: Forward: 5′ CTTCTGGGCTCAAGTGATCCT 3′; Reverse: 5′ TGTGCCATGAGACTCCATCAG 3′. Human GAPDH: Forward: 5′ GATCATCAGCAATGCCTCCT 3′; Reverse: 3′ TGTGGTCATGAGTCCTTCCA 5′. Primer efficiency and specificity was confirmed by dissociation curve analysis, which showed a single, sharp peak, indicating amplification of one specific target (Supplemental Figure S1). The Ct values for GAS5 ranged between 24.0 and 27.0. For absolute quantification, a standard curve was generated. cDNA concentration was measured on Nanodrop and amounts ranging from 100–0.1 ng were used to correlate quantity with the threshold cycle (Ct) number. A linear relationship (r^2^ = 0.998) was obtained for GAS5. RT-qPCR was then performed on samples and standards in triplicate. The plate setup also included a standard series, no template control, no RNA control, no reverse transcriptase control, and no amplification control. The dissociation curve was analyzed for each sample. Absolute quantification (AQ) of GAS5 expression in individual samples was calculated by normalizing to GAPDH.

### 2.4. Acute TCDD Exposure in Mice

Male and female 3-month-old C57BL/6J mice were purchased from Jackson laboratory. All experiments and procedures were approved by the James A. Haley Veterans’ Hospital and University of South Florida Institutional Animal Care and Use Committee (IACUC) and were consistent with the governing guidelines and recommendations of AWA and HREA. All experiments complied with the ARRIVE guidelines. Animals were raised and studied in a pathogen-free environment, housed in plastic, sawdust-covered cages with normal light–dark cycle and free access to chow and water. A total of 24 mice (12 male and 12 female) were included in the study. Prior to treatment, a few drops of blood were collected via lancet for analysis of Gas5 via direct blood qPCR (methods below) and glucose levels via a glucometer for baseline measurements. Six mice (3 male and 3 female) were randomly assigned to each 2,3,7,8-tetrachlorodibenzo-p-dioxin (TCDD) exposure group. Mice received a single subcutaneous injection of 0 μg (control), 0.046 μg, 0.46 μg, or 4.6 μg TCDD. One week after TCDD exposure, blood was collected via lancet for analysis of Gas5 via direct blood qPCR and glucose levels via a glucometer.

### 2.5. Gas5 Direct Blood qPCR for Mouse Samples

Whole blood (3 µL) collected from mice was mixed with 1mL nuclease-free water containing RNase Out and incubated at room temperature for 15 min, followed by centrifugation at 10,000 rpm for 2 min. The supernatant was discarded. 200 μL of InstaGene Matrix (Bio-Rad, Hercules, CA, USA, catalog #732-6030) was added to the pellet and incubated at 95 °C for 15 min, followed by centrifugation at 10,000 rpm for 2 min. 8 µL of the resulting supernatant was used as the template in a 20 μL qPCR reaction containing 5 µL Lyo-Ready™ Direct one-step RT-qPCR Blood master mix (Meridian Bioscience, Cincinnati, OH, USA, catalog #MDX123), 1 μL of Gas5 primers, and 1 µL SYBR^®^ Green Fluorescent DNA Stain (MiTeGen, Ithica, NY, USA, catalog #M-PCR-378), per manufacturer’s instructions. RT-qPCR was performed on the ViiA 7 Real-Time PCR System (Applied Biosystems, Carlsbad, CA, USA). Samples were run in triplicate with a standard series, no template control, and no reverse transcriptase control. A standard curve was generated for each target and used to calculate the AQ of target expression, which was then normalized to RPLP0 expression. Mouse Gas5 primers: Forward: 5′ CTCCTGTGACAAGTGGAC 3′; Reverse: 5′ AACACAATATATCTGACACCATC 3′. Mouse RPLP0 primers: Forward: 5′ GACATGCTGCTGGCCAATAAG 3′; Reverse: 3′ CAACATGTTCAGCAGTGTG 5′.

### 2.6. Gas5 from Mouse DBS

Blood was collected from six 4 months old C57BL/6J mice. From each mouse, 150 μL was collected into an anticoagulant containing tube combined with 500 μL of RNAlater. RNA was extracted as described above for human samples. Separately, 10 μL of blood was collected from the same mouse on Whatman FTA paper and dried at 22 °C for 4 hours, then stored at −80 °C for 1 week. RNA was extracted from three 3mm punches as described above for the human DBS samples. RNA quality was evaluated by TapeStation. Results showed a RIN of 8.5 for DBS and a RIN of 8.6 for blood. 250 ng RNA from DBS or plasma was used to synthesize cDNA via BioRad iScript kit (BioRad, Hercules, CA, USA, catalog #1708841). RT-qPCR was performed in triplicate with 2X Maxima SYBR Green Mastermix with ROX (Thermo Scientific, Waltham, MA, USA, catalog A25742) on an Applied Biosystem’s Viia7 RT-qPCR machine, using 1 μL cDNA per reaction. Samples were run in triplicate with a standard series, no template control, and no reverse transcriptase control. A standard curve (Supplemental Figure S2) was generated for each target and used to calculate the AQ of target, which was then normalized to RPLP0 expression. A linear relationship (r^2^ = 0.997) was obtained for Gas5. Mouse Gas5 and RPLP0 primers were the as those described above in Section 2.5.

### 2.7. Statistical Analysis

Experimental samples were run in triplicate. The multivariate model included 179 participants (participants with missing data were excluded from this analysis) and was statistically significant overall (χ^2^(7) = 103.46, *p* < 0.001). Gender, diabetes status, and obesity status were not statistically significant predictors in the robust model. Human DBS data were not normally distributed and were therefore analyzed using Spearman’s rho correlation, or Mann-Whitney U-test for bivariate analysis. The relationship between GAS5 and diabetes status was investigated using ROC curve analysis to report the area under the curve (AUC) with 95% confidence intervals (CIs). A generalized linear model with a normal distribution, an identity link, and robust covariance estimation was used to account for variance heterogeneity and analyze the relationship between GAS5 and war era, adjusting for age, gender, Agent Orange exposure, and diabetes status among Veterans. Mouse data were found to be normally distributed via Shapiro-Wilk and Kolmogorov-Smirnov testing. Subsequent analyses were completed using One-Way ANOVA, Unpaired Student’s t-test, and Pearson’s coefficient where indicated. The statistical analysis was conducted by SPSS v.30 Software and GraphPad Prism v.10.0.0 for Windows (GraphPad Software, Boston, MA, USA). *p* values less than 0.05 indicated statistically significant.

## 3. Results

### 3.1. Participant Characteristics

The JAHVH Research Biospecimen Repository collected dried blood spots (DBS) from 186 participating Veterans. As shown in Table 1, samples were obtained from Veterans who were deployed during the Vietnam War (VW; *n* = 102), the Gulf War (GW; *n* = 34) or other war eras (*n* = 50). Veterans deployed during VW, GW, and other (non-VW/GW) conflicts were on average 67.1, 55.5, and 71.8 years of age, respectively. The samples were predominantly from male Veterans (89.7%, *n* = 166) compared with female Veterans (10.3%, *n* = 19). Gulf War Veterans had the greatest proportion of female Veterans (23.5%, *n* = 8) compared with VW or other war era Veterans. Most DBS samples were obtained from white, non-Hispanic identifying Veterans across all war eras (total 82.1%, *n*= 152).

### 3.2. Older Age Was Independently Associated with Lower GAS5 Levels

**Figure 1:**
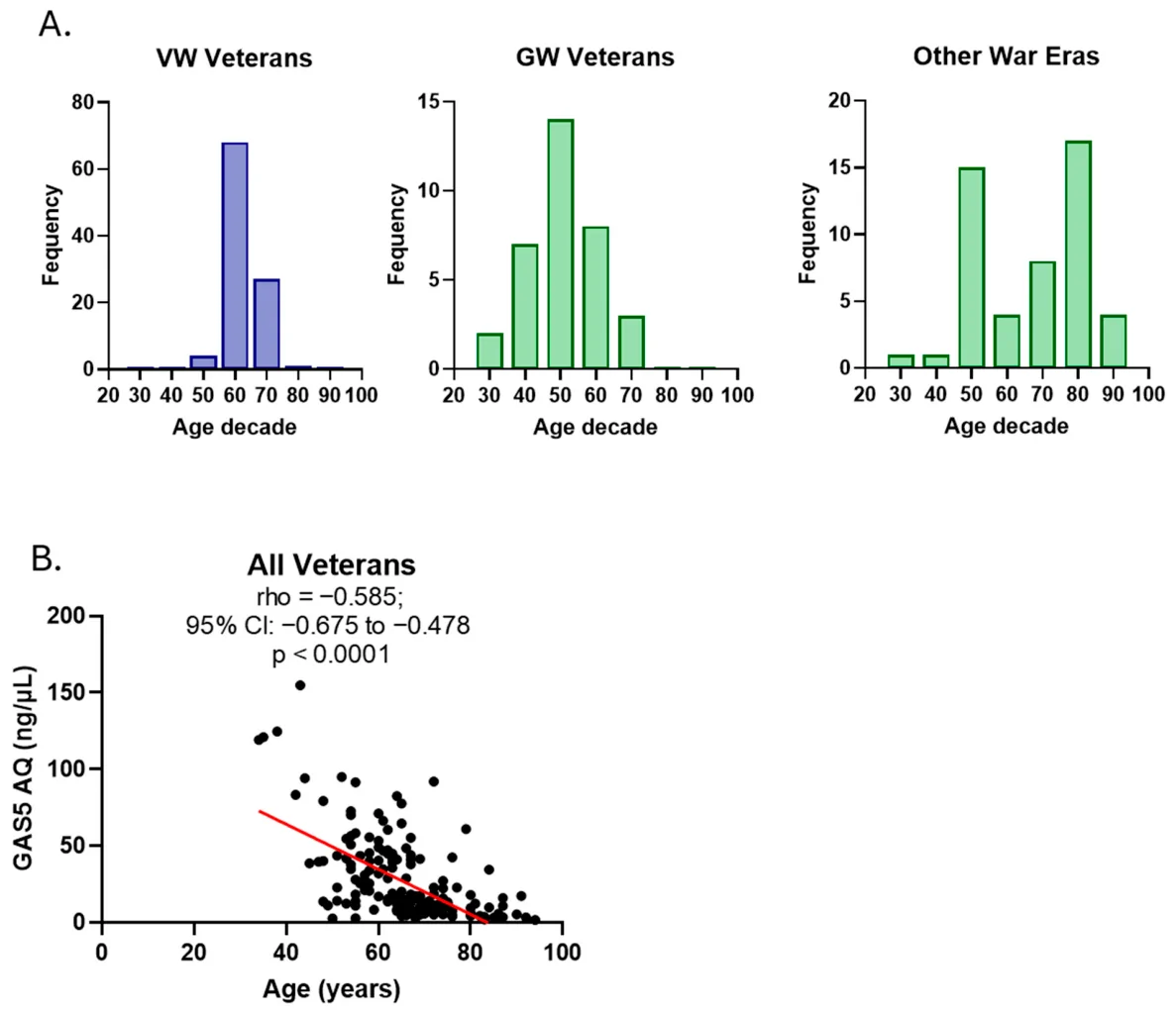
(**A**) Histograms of Veteran ages at the time of blood collection analyzed by war era. (**B**) GAS5 levels were analyzed by RT-qPCR using dried blood spots from Veterans and absolute quantities (AQ) was calculated from the standard curve. Spearman’s rho shows negative correlation of GAS5 AQ value (ng/μL) vs. Veterans’ age (years). N = 186; rho= −0.585, 95% confidence interval (CI): −0.675 to −0.478; *p* < 0.0001. Black dots represent each individual Veteran and their blood GAS5 level compared to their age at blood collection. The red line represents the simple linear regression fir to the points on the graph.

We evaluated the age distribution of Veterans from each war era (VW, GW, Other; Figure 1A). GW Veterans had a median age of 54 years (min: 34, max: 76) with the largest proportion of this group (41%) being in their 50s. VW Veterans had a median age of 67 years (min: 58, max: 80); almost 70% were in their 60s. Veterans of other (non-VW/GW) war eras had a median age of 75 years (min: 33, max: 93), with 42% older than 80 years of age.

RT-qPCR was performed on DBS samples from this cohort to quantify lncRNA GAS5. Results demonstrate that lncRNA GAS5 can be measured robustly from the DBS samples. Next, we analyzed the correlation between GAS5 levels and age at time of collection. Results show GAS5 levels declining with age (Figure 1B), a statistically significant, negative correlation. Multivariate analysis showed that older age was independently associated with lower GAS5 AQ, with each additional year of age corresponding to a 1.35 ng decrease in GAS5 AQ. A generalized linear model was fit to examine age and GAS5 AQ. Age at collection was a significant negative predictor of GAS5 AQ (*B* = −1.35, 95% CI −1.75, −0.95, Wald χ^2^(1) = 44.01, *p* < 0.001), indicating that GAS5 AQ decreased with increasing age.

### 3.3. GAS5 Levels in DBS Are Negatively Correlated with Diabetes and A1C Values

**Figure 2:**
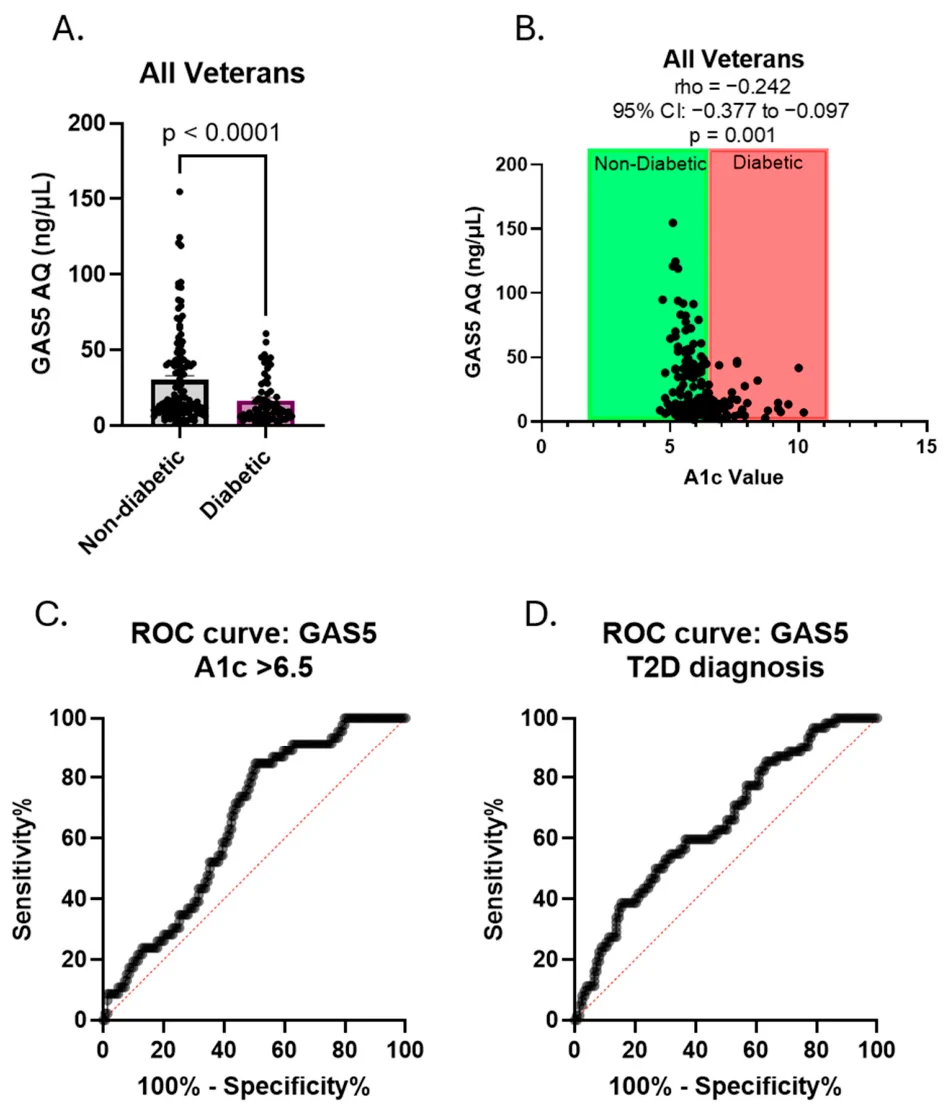
(**A**) GAS5 AQ in diagnosed diabetic and non-diabetic Veterans from all war eras *n* = 62 (diabetic), 124 (non-diabetic). Analyzed by Mann-Whitney test; *p* < 0.0001 (**B**) Spearman’s rho shows negative correlation of GAS5 AQ to A1c values in all war era Veterans (N = 186). Rho = −0.242, 95% CI: −0.377 to −0.097; *p* = 0.001 (**C**) Receiver Operating Curve (ROC) was performed on GAS5 levels for A1c values > 6.5. Graph shows area under curve (AUC): 0.6537,95% CI: 0.5714 to 0.7361; *p* = 0.0018. The red dashed line represents the random classifier while the black line represents the calculated classifier for the data. (**D**) ROC curves was performed on GAS5 levels for T2D diagnosis shows AUC: 0.6542,95% CI: 0.5724 to 0.7360; *p* = 0.0006. The red dashed line represents the random classifier while the black line represents the calculated classifier for the data.

Given prior evidence linking GAS5 to T2D prevalence, we investigated the relationship between GAS5 levels and diabetes diagnosis or A1c values in this Veteran cohort. Results (Figure 2A) show that Veterans with a T2D diagnosis had significantly lower GAS5 levels than non-diabetic Veterans. Since A1c values (> 6.5 indicates diabetes) reflect the average blood glucose over the 3 months prior to DBS sampling, we analyzed GAS5 levels alongside A1c values. Spearman’s rho analysis showed a highly significant negative correlation between GAS5 levels and A1c values (rho= −0.242, 95% CI: −0.377 to −0.097; *p* = 0.001; Figure 2B). Next, we generated exploratory receiver operating characteristic (ROC) curves. ROC analysis by A1c values (> 6.5) revealed a significant area under the curve (AUC) (AUC: 0.6537, 95% CI: 0.5714 to 0.7361; *p* = 0.0018; Figure 2C). The optimal cut off, as determined by Cutoff finder website, was GAS5 AQ of 14.42 with 71.7% sensitivity. ROC analyzed by T2D diagnosis status showed similar results (AUC: 0.6542; 95% CI: 0.5724 to 0.7360; *p* = 0.0006; Figure 2D). Overall, these results confirm a significant association between A1c values or T2D and GAS5 in this Veteran population.

### 3.4. VW Veterans Have Lower GAS5 Levels Compared to Veterans from Other War Eras

**Figure 3:**
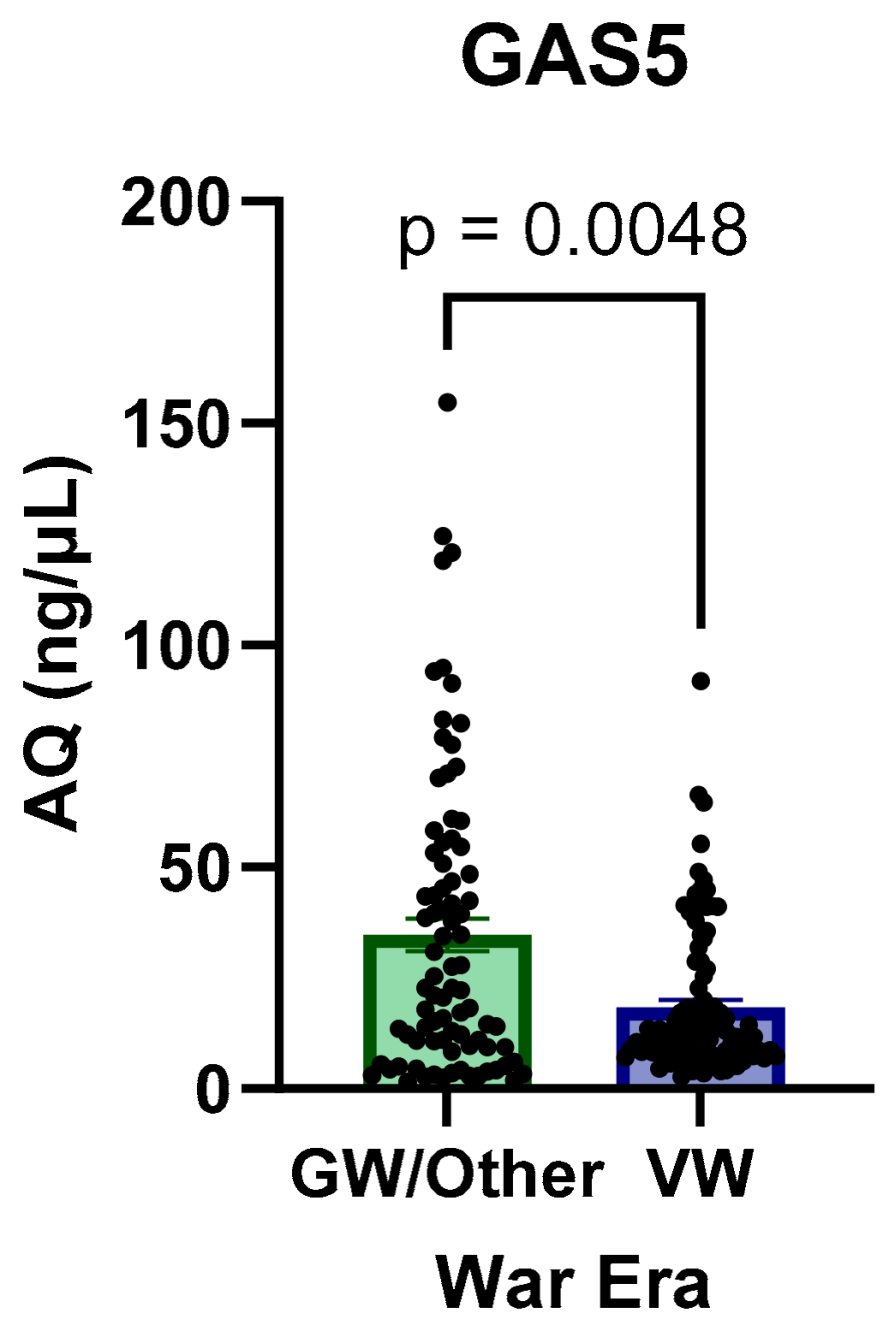
GAS5 AQ was evaluated by War Era sorted as GW/Other wars (combined total *n* = 84) and VW (*n* = 102). Statistical analysis performed using Mann-Whitney test, *p* = 0.0048.

Next, we analyzed GAS5 levels by the war era in which the US Veterans served, grouped as Vietnam War (VW), the Gulf War (GW) or other wars (non-VW/GW). VW Veterans had, on average, lower blood GAS5 levels than GW and other war era Veterans combined (Figure 3). The average GAS5 AQ in non-VW Veterans was 34.7 ng whereas that of VW Veterans was 18.5 ng, a nearly 2-fold difference in expression. A generalized linear model was fit to examine predictors of GAS5 AQ (ng). The multivariate analysis showed that War era was significantly associated with GAS5 AQ (Wald χ^2^(2) = 11.84, *p* = 0.003). Compared with the other war-era group, VW-era participants had significantly lower GAS5 AQ. (B = −10.927, 95% CI: −17.943 to −3.912, *p* = 0.002).

### 3.5. Vietnam War Veterans Have a Higher Incidence of T2D Concurrent with Lower Blood GAS5 Levels

**Figure 4:**
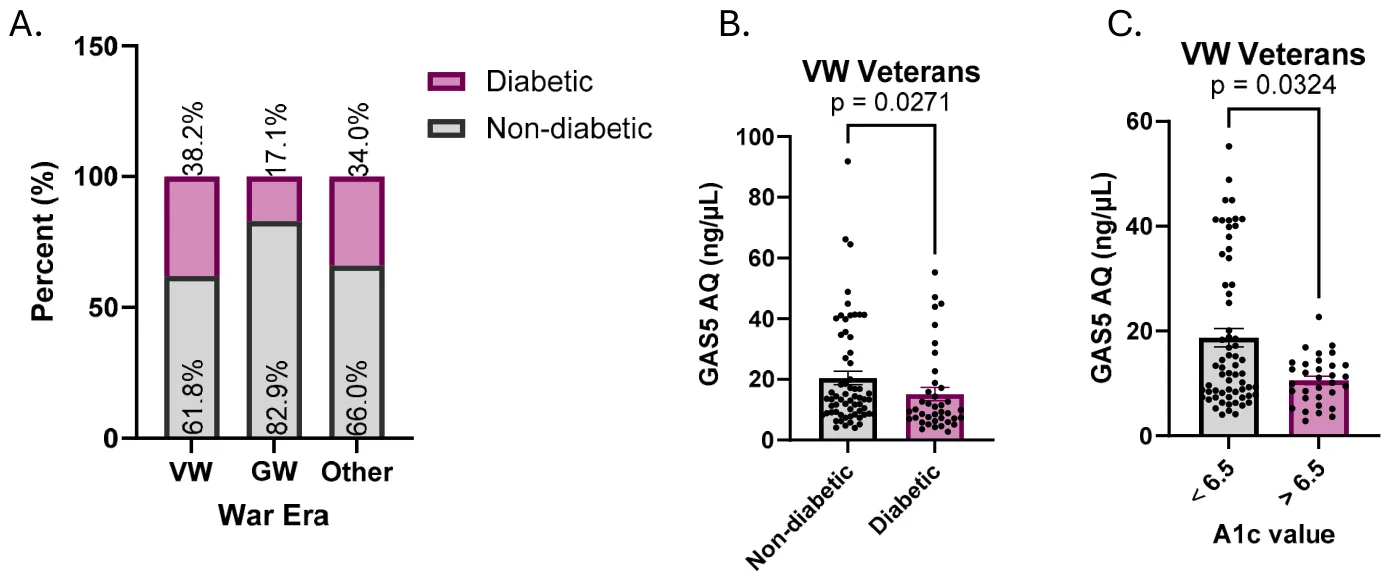
(**A**) Bar graph of percent of Veterans from each war era that were diagnosed with diabetes. (**B**) GAS5 AQ in Vietnam War (VW) Veterans diagnosed diabetic or non-diabetic. Analyzed by Mann-Whitney test; *p* = 0.0271. (**C**) GAS5 AQ in Vietnam War (VW) Veterans versus A1c < 6.5 or A1c > 6.5. Analyzed by Mann-Whitney test; *p* = 0.0324.

In this cohort, 38.2% of VW Veterans, 17.1% of GW Veterans, and 34.0% Veterans of other war eras were diabetic (Chi-square: 11.92; df: 2; *p*: 0.0026; Figure 4A). Since the VW Veterans in our cohort had the highest incidences of diabetes, we further analyzed their samples (Figure 4B). GAS5 levels were significantly lower in VW Veterans with T2D diagnosis codes in their charts. Analysis of GAS5 levels against A1c values at time of blood collection likewise showed significantly lower GAS5 levels among individuals with A1c > 6.5 (Figure 4C).

### 3.6. Low GAS5 Levels in VW Veterans Are Independent of BMI

**Figure 5:**
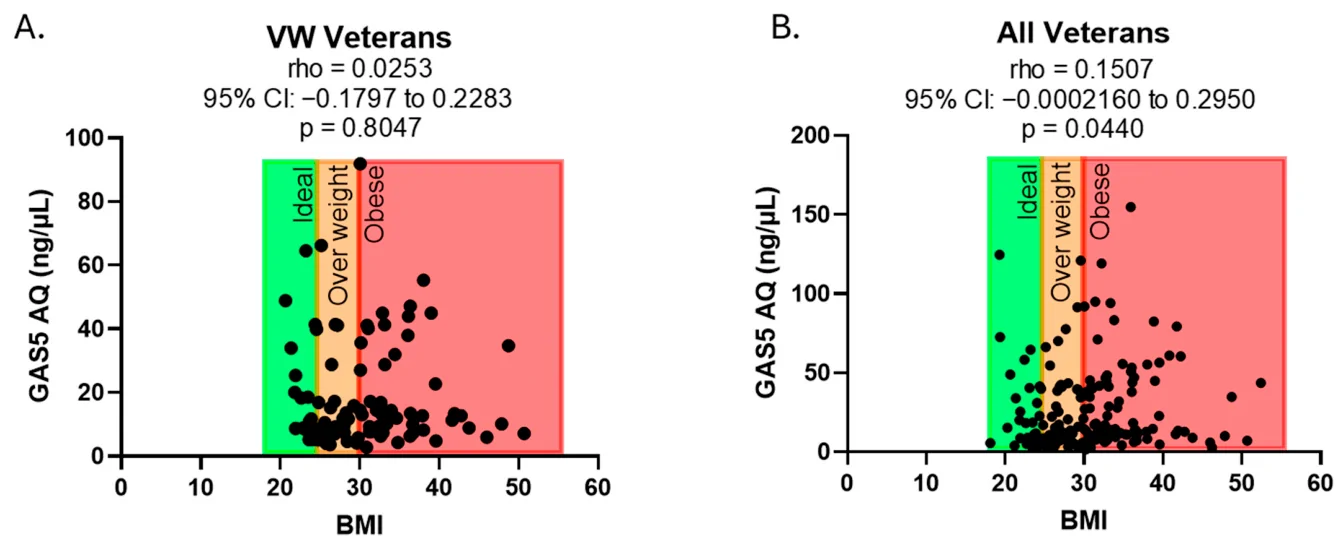
(**A**) Spearman’s rho correlation of GAS5 AQ to BMI in VW Veterans (*n* = 101) rho = 0.0253, 95% confidence interval (CI: −0.179 to −0.228); *p* = 0.804. (**B**) Spearman’s rho correlation of GAS5 AQ to BMI in all war era Veterans; *n* = 179; (7 samples were dropped due to incomplete data).

Since there is a known connection between BMI > 30 and A1c values, we also examined the correlation between GAS5 levels and BMI. Results show no correlation between GAS5 levels and BMI in VW Veterans (Figure 5A), but a weak correlation in all Veterans. (Figure 5B). A generalized linear model showed no association between obesity status (BMI > 30) and outcome (*B* = 0.00, 95% CI −6.18, 6.18, Wald χ^2^(1) = 0.00, *p* = 1.000).

### 3.7. A Small Subset of VW Veterans with Self-Reported Exposure to Agent Orange (AO) Have Lower Blood GAS5 Compared to Other VW Veterans

**Figure 6:**
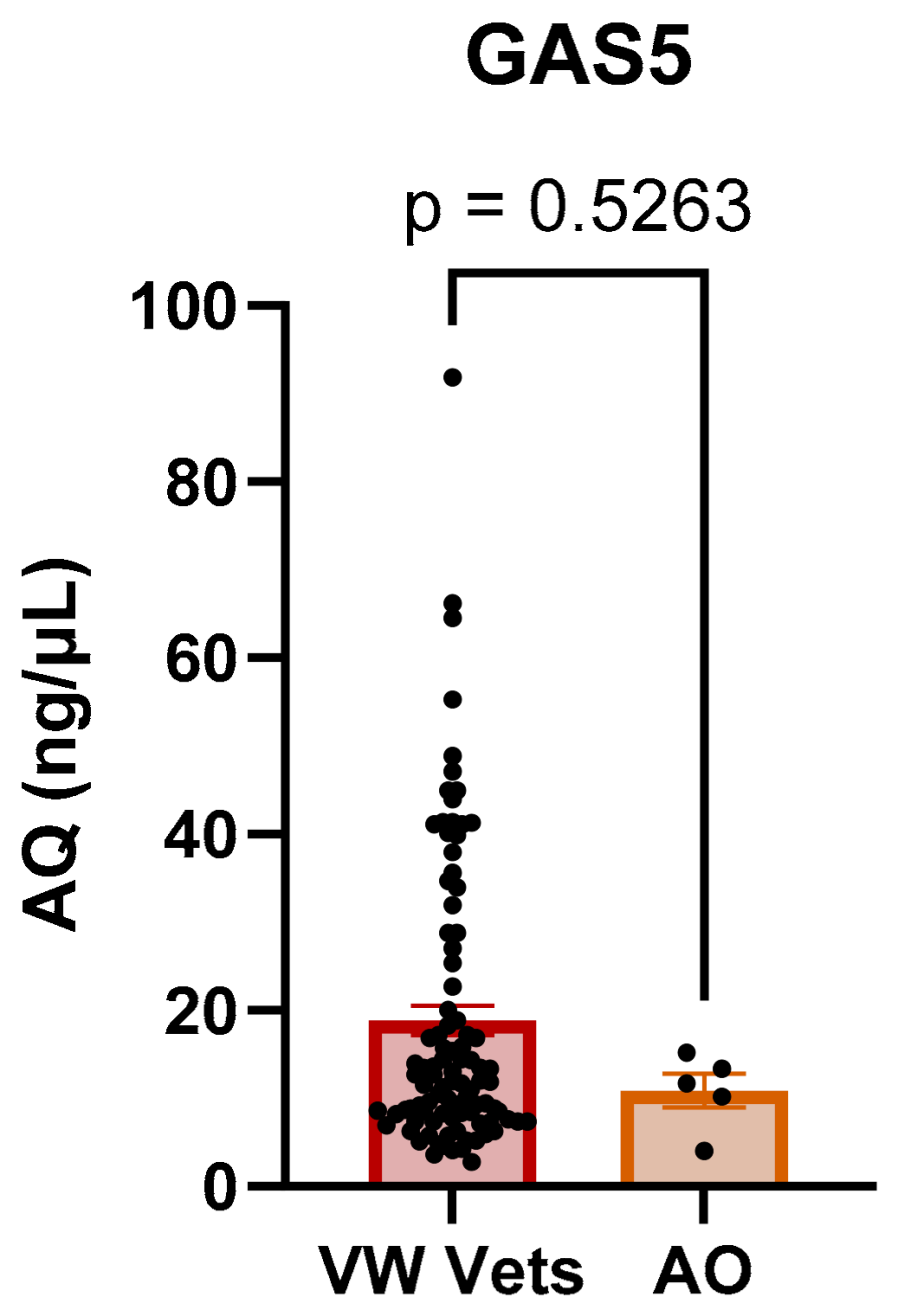
GAS5 AQ in VW Veterans that did not (*n* = 97) or did (*n* = 5) report exposure to AO. Analyzed by Mann-Whitney test, *p* = 0.5263.

One factor distinguishing VW Veterans from Veterans of other war eras is the exposure to the herbicide Agent Orange (AO). We evaluated the correlation of GAS5 levels and self-reported AO exposure in VW Veterans. Results show lower GAS5 levels in the Veterans who self-reported AO exposure (Figure 6). Non-exposure to Agent Orange was associated with higher GAS5 AQ than exposure (*B* = 10.332, 95% CI: 4.178 to 16.485, *p* < 0.001). However, this finding should be interpreted with caution as only 5 participants in this cohort had reported Agent Orange exposure. None of these individuals were being treated for diabetes at the time the DBS samples were collected. However, one Veteran who reported handling AO had one of the highest A1c values in this study (9.6) and three of these Veterans had A1c values indicative of pre-diabetes (5.7–6.4).

### 3.8. Acute Exposure to Dioxin in Mice Shows a Dose-Dependent Decline in Circulating Gas5 Concurrent with Increased Blood Glucose

**Figure 7:**
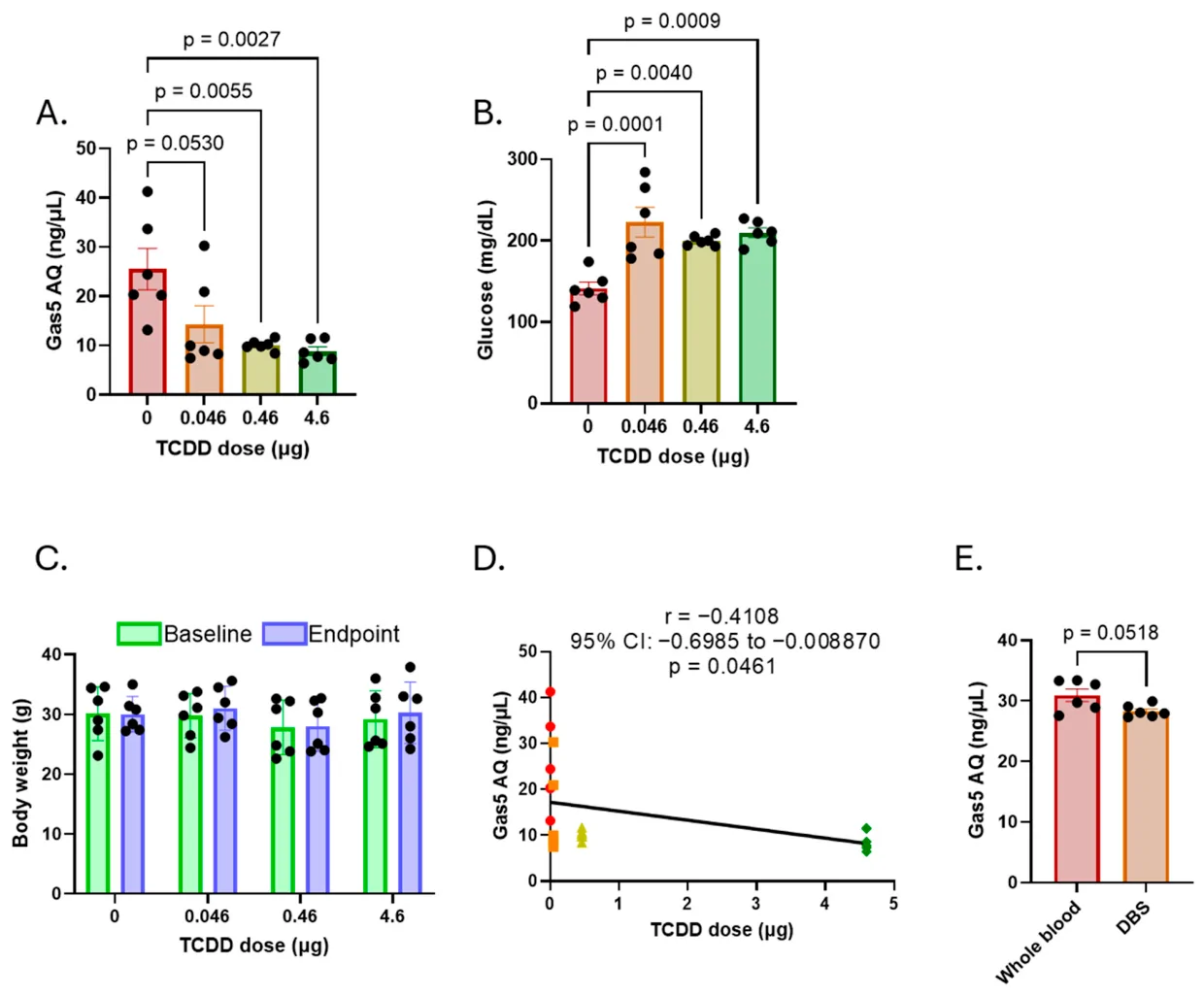
An equal number of male and female C57BL/6J mice were exposed to either 0, 0.046, 0.46, or 4.6 μg TCDD once via subcutaneous injection. Blood was collected one week later for analysis of Gas5 and glucose levels. (**A**) Gas5 was measured by direct blood qPCR. Gas5 AQ for mice exposed to each TCDD dose. *n* = 6. Analyzed by One-way ANOVA. (**B**) Glucose levels were measured by glucometer. Measured blood glucose levels for mice exposed to each TCDD dose. *n* = 6. Analyzed by One-way ANOVA. (**C**) Body weights were measured at baseline and the endpoint to assess whether changes in blood glucose were attributable to weight gain. *n* = 6. (**D**) 0 μg (Red dot), 0.046 μg (Orange square), 0.46 μg (yellow triangle), or 4.6 μg (green diamond) TCDD are indicated on the graph, N = 24. Correlation of Gas5 AQ and TCDD dose analyzed by Pearson’s correlation coefficient r = −0.4108; 95% CI: −0.6985 to −0.009; *p* = 0.0461. (**E**) Comparison of Gas5 levels in mouse whole blood versus dried blood. *n* = 6. Analyzed by Student’s unpaired *t*-test; *p* = 0.0518.

Since the AO-exposure analysis (Figure 6) was based on only five self-reported cases, we sought to evaluate this observation further using an animal model. During the VW, the herbicide AO was contaminated with the highly toxic endocrine disrupting chemical 2,3,7,8-tetrachlorodibenzo-p-dioxin (TCDD). We evaluated whether TCDD exposure affects Gas5 levels in vivo using a mouse model with acute TCDD exposure, measuring blood Gas5 and glucose levels. Mice received a single injection of TCDD at 0 μg (no exposure), 0.046 μg, 0.46 μg, 4.6 μg. One week after exposure, blood Gas5 levels were measured using a rapid, blood direct RT-qPCR method optimized in our laboratory. The glucose levels were measured using a droplet glucometer. TCDD produced a significant dose-dependent decrease in circulating Gas5 levels (Figure 7A). Blood glucose increased with TCDD exposure and plateaued (Figure 7B). The decrease in Gas5 levels was independent of body weight (Figure 7C); there were no significant changes in body weight at the experimental baseline and endpoint, nor any changes in feeding or grooming behavior. These results show that the TCDD doses were not detrimental to the mice. Gas5 levels and TCDD dose showed a significant negative correlation (Figure 7D).

Next, we compared Gas5 levels using paired DBS and blood from the same mice. Blood was processed and RNA isolated on the same day. DBS was stored at −80°C for 1 week before RNA was isolated. RT-qPCR was performed on these samples to measure Gas5 levels. Results demonstrate comparable AQs of Gas5 between DBS and blood (Figure 7E).

## 4. Discussion

This study demonstrates the feasibility of using DBS to measure levels of lncRNA GAS5 in a cohort if US Veterans. Older age was independently associated with lower GAS5 levels. Older age is associated with reduced insulin secretion concurrent with increased insulin resistance as a result of chronic oxidative stress during aging [20,21]. When stratified by war era, T2D was more prevalent among VW Veterans than among GW or other war era Veterans, concurrent with low GAS5 in VW Veterans. A1c values, which are used to diagnose T2D (A1c > 6.5 indicates diabetes), were significantly negatively correlated with blood GAS5 levels in VW Veterans. Since obesity is a predominant risk factor for T2D, we additionally evaluated the correlation between GAS5 levels and BMI and found no significant correlation in our Veteran cohort.

Deployment-related exposures represent another factor that could influence both GAS5 levels and T2D development. During the Vietnam War, the herbicide Agent Orange (AO) was widely used to clear vegetation in Vietnam, Laos and Cambodia to reduce foliage camouflage used by the Viet Cong forces [22]. AO is a 1:1 mixture of 2,4-dichlorophenoxyacetic acid (2,4-D) and 2,4,5-trichlorophenoxyacetic acid (2,4,5-T). However, a byproduct of 2,4,5-T is the dioxin 2,3,7,8-tetrachlorodibenzo-p-dioxin (TCDD) which is a human endocrine disrupting chemical [22]. During Operation Ranch Hand, over 11 million gallons of AO were sprayed aerially by C-123 aircrafts [23] exposing nearby ground troops. Personnel who handled and dispersed AO were heavily exposed due to the minimal protective measures at the time. TCDD exposure has been shown to cause an insulin resistant state [24,25,26], while longitudinal studies have linked Ranch Hand Veterans with both diabetes [23] and high TCDD levels in serum [27]. Diabetes is now a presumptive condition for VW Veterans [28].

AO exposure has been linked to a range of negative health outcomes including multiple cancers (e.g., soft tissue sarcoma and non-Hodgkin lymphoma), birth defects, and diabetes [23]. Michalek and Pavuk [23] found an increased risk of T2D development among Operation Ranch Hand Veterans who experienced 90 days or more of AO spraying and who served before or during 1969. Herbicides were first used during Operation Ranch Hand in 1962. AO, specifically, was sprayed from 1965 to 1970 [22]. Similar results were observed by Hendriksen et al. [29] in their study of Ranch Hand Veterans. They found that glucose abnormalities, diabetes diagnosis, and the use of oral diabetic medications all increased with higher blood dioxin levels. Further, Veterans with high levels of blood dioxin developed diabetes earlier than those with lower levels [28]. In the present study, five Veterans reported AO exposure; none were receiving treatment for T2D at the time of DBS collection (2014). However, these samples showed significantly lower GAS5 expression than samples from non-exposed Veterans. It is possible that many of the VW Veterans were exposed to AO indirectly and without their knowledge at some point during their deployment, which may help explain the significantly lower GAS5 levels observed broadly among this group.

Given the very small sample size of self-reported AO-exposed Veterans and the risk of self-report bias, we used a TCDD exposure mouse model to test more directly whether TCDD reduced Gas5 levels. In doing so, we established a rapid blood test for measuring Gas5 levels. In mice, TCDD injection produced a decline in circulating gas5 levels concurrent with increased blood glucose, independent of weight changes. These results indicate that Gas5 is a target of TCDD and show a negative correlation between Gas5 and TCDD exposure dose. Since previous studies have shown that low Gas5 is causal to T2D, and that GAS5 regulates insulin receptor signaling and glucose uptake, these results suggest a possible link between TCDD-induced GAS5 depletion and acute hyperglycemia. However, a long-term study is required to establish a diabetes diagnosis concurrent with TCDD-induced low Gas5 levels to evaluate the chronic long term AO exposure reported in VW Veterans. Our results also demonstrate comparable measurement of Gas5 from DBS and blood samples from mice.

Limitations: Future work should prioritize the collection of additional samples from Veterans who self-report handling of AO or have confirmed AO exposure, to increase statistical power. In the mouse study, the response to TCDD was evaluated after 1 week of exposure, indicating hyperglycemia but not necessarily insulin resistance. A longitudinal mouse model with chronic TCDD exposure over several months is currently being evaluated to better model TCDD exposure effects in the Veteran population. Additional testing for diabetes (insulin levels, glucose tolerance, insulin sensitivity) will be needed to clarify the mechanistic relationship between TCDD exposure, GAS5 levels, and diabetes pathology. The molecular mechanisms underlying the cause of TCDD-mediated decline in Gas5 are being pursued in our laboratory.

## 5. Conclusions

This study demonstrates our ability to measure lncRNA GAS5 in DBS, an approach which offers the advantages of requiring lower volumes of blood samples (drops from finger pricks), ease of storage and transport (short-term transport at room temperature). These finding show that lncRNA blood biomarkers can be robustly measured in DBS supporting the use of DBS in longitudinal studies of chronic diseases among active-duty service members or Veterans with toxic exposures during military service. More broadly, DBS collection could be incorporated for Veterans and civilians in remote locations away from health clinics to facilitate the detection of genetic biomarkers for diagnosis and prognosis of chronic diseases.

## 6. Patents

US Patent 10,724,097 awarded to N.A.P. is not yet licensed.

## Figures and Tables

**Table 1:** Demographics of study population.

	Study Group		
	VW Veterans	GW Veterans	Other Veterans
Characteristic	(*n* = 102)	(*n* = 34)	(*n* = 50)
**Age, mean (SD) (year)**	67.1 (4.8)	55.5 (9.8)	71.8 (15.0)
**Sex, no (%)**			
Male	97 (95.1)	26 (76.5)	44 (88.0)
Female	5 (4.9)	8 (23.5)	6 (12.0)
**Racial background, no (%)**			
White/Non Hispanic	87 (85.3)	25 (73.5)	41 (82.0)
White/Hispanic	3 (2.9)	4 (11.8)	3 (6.0)
White/Unknown	2 (2.0)	0 (0.0)	1 (2.0)
Black/Non Hispanic	6 (4.9)	3 (8.8)	3 (6.0)
Black/Hispanic	1 (1.0)	1 (2.9)	1 (2.0)
Unknown/Hispanic	1 (1.0)	1 (2.9)	0 (0.0)
Pacific Islander	1 (1.0)	0 (0.0)	0 (0.0)
Asian	1 (1.0)	0 (0.0)	0 (0.0)
American Indian	0 (0.0)	0 (0.0)	1 (2.0)
**Instance of diabetes (A1c > 6.5), no (%)**			
Male	35 (34.3)	3 (8.8)	3 (6.0)
Female	0 (0.0)	1 (2.9)	1 (2.0)
**A1c value, mean (SD)**			
Male	6.3 (1.1)	6 (1.0)	6.1 (0.9)
Female	5.7 (0.2)	5.65 (0.5)	6 (0.7)

## Data Availability

All data are shown in the manuscript.

## Supplementary Materials

The following supporting information can be downloaded at: https://www.mdpi.com/article/10.3390/biology15171488/s1, Figure S1: Human GAS5 standard curve and melt curve plot; Figure S2: Mouse Gas5 standard curve and melt curve plot.

## Abbreviations

The following abbreviations are used in this manuscript:

VW	Vietnam War
GW	Gulf War
AO	Agent Orange
T2D	Type 2 Diabetes
GAS5	growth-arrest specific transcript 5
lncRNA	Long non-coding RNA
DBS	Dried blood spot
TCDD	2,3,7,8-tetrachlorodibenzo-p-dioxin
AQ	Absolute quantification
