# Supplementary material for: Dried Blood Spot-Based Quantification Indicates Correlation Between lncRNA GAS5, Diabetes, Age, and War Eras in US Veterans
Source: Biology (Basel). 2026 Sep 2;15(17):1488. doi: 10.3390/biology15171488 (PMC13565461; doi:10.3390/biology15171488)

Supplemental Figure S1: Human GAS5 standard curve and melt curve plot

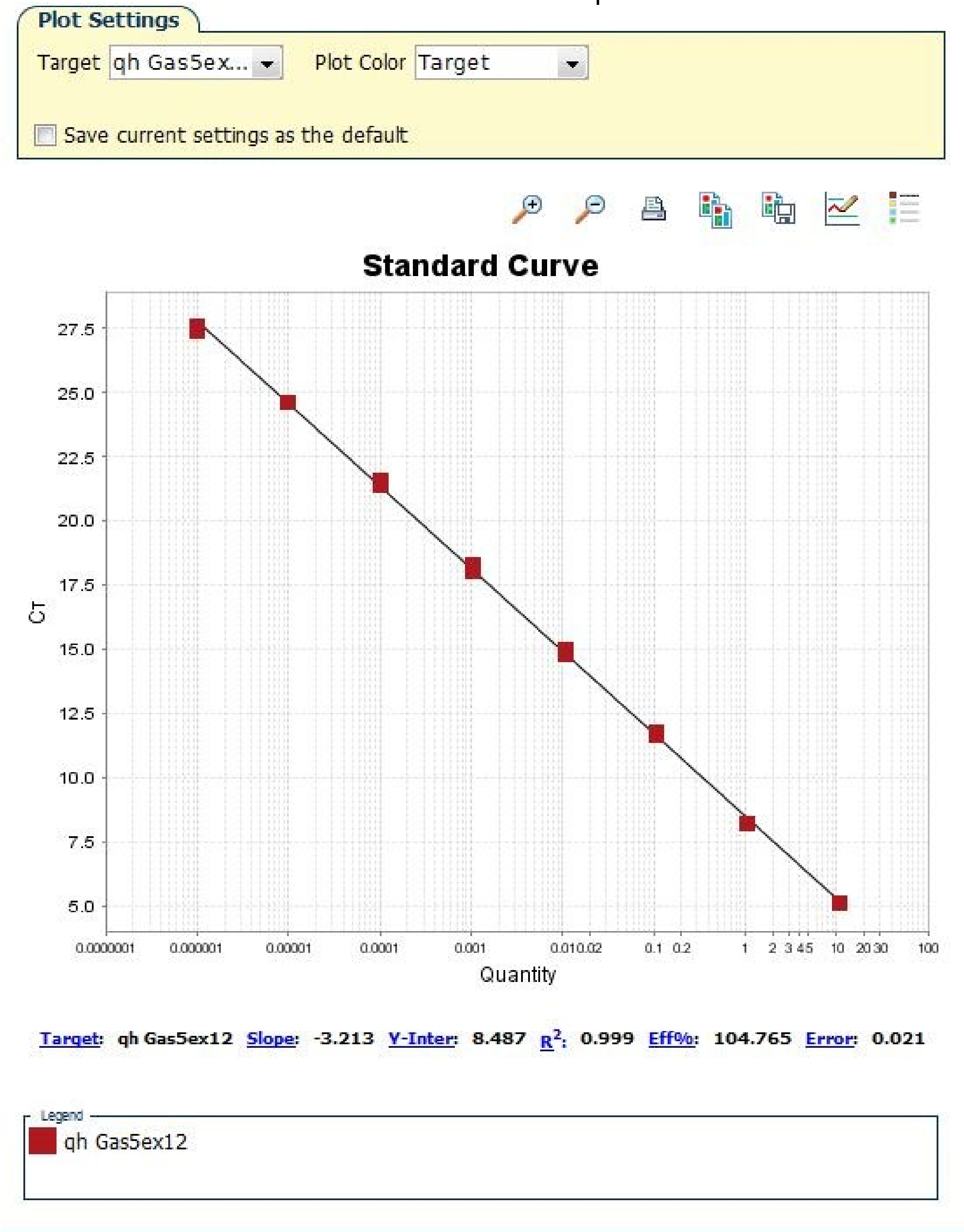

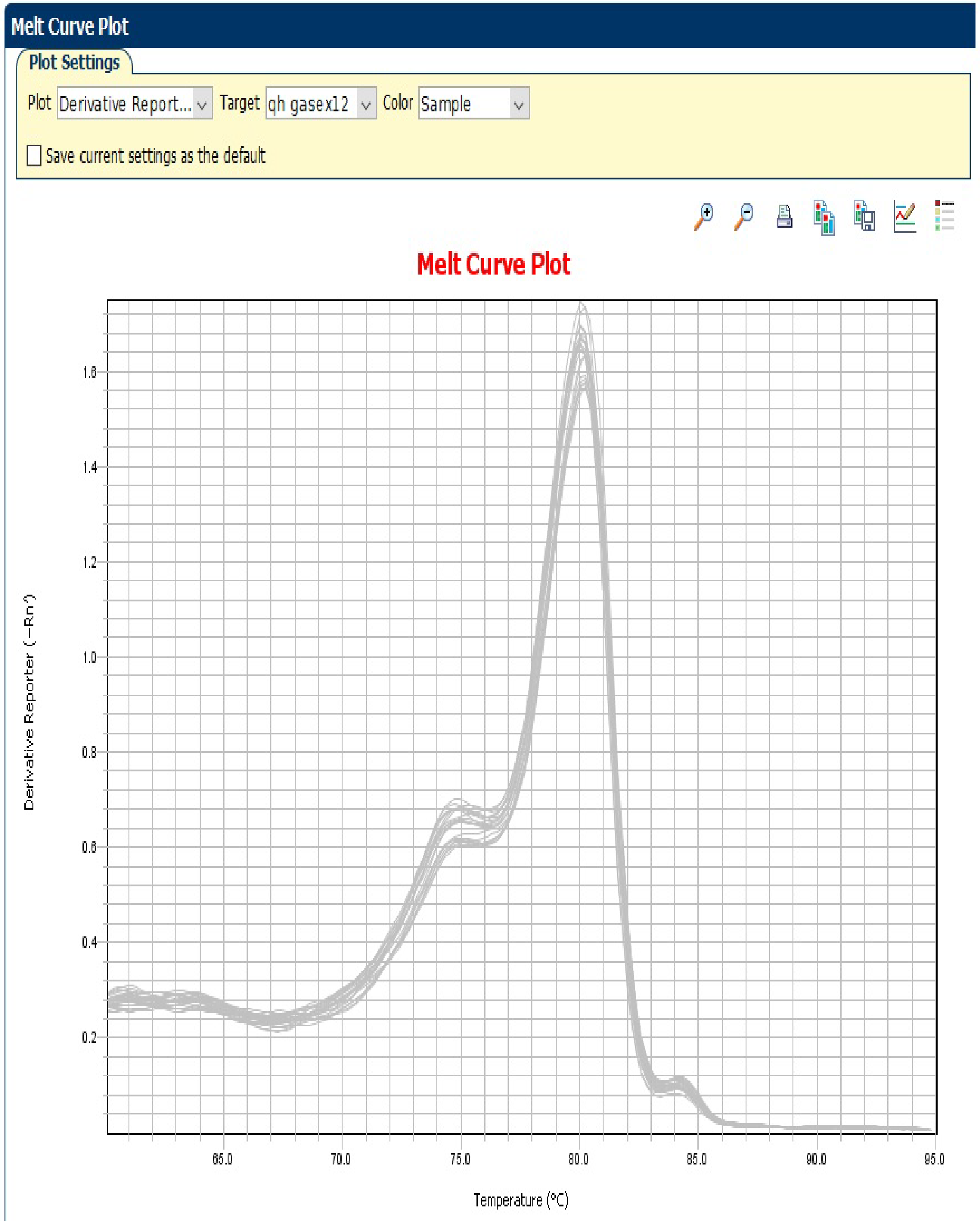

Supplemental Figure S2: Mouse Gas5 standard curve and melt curve plot

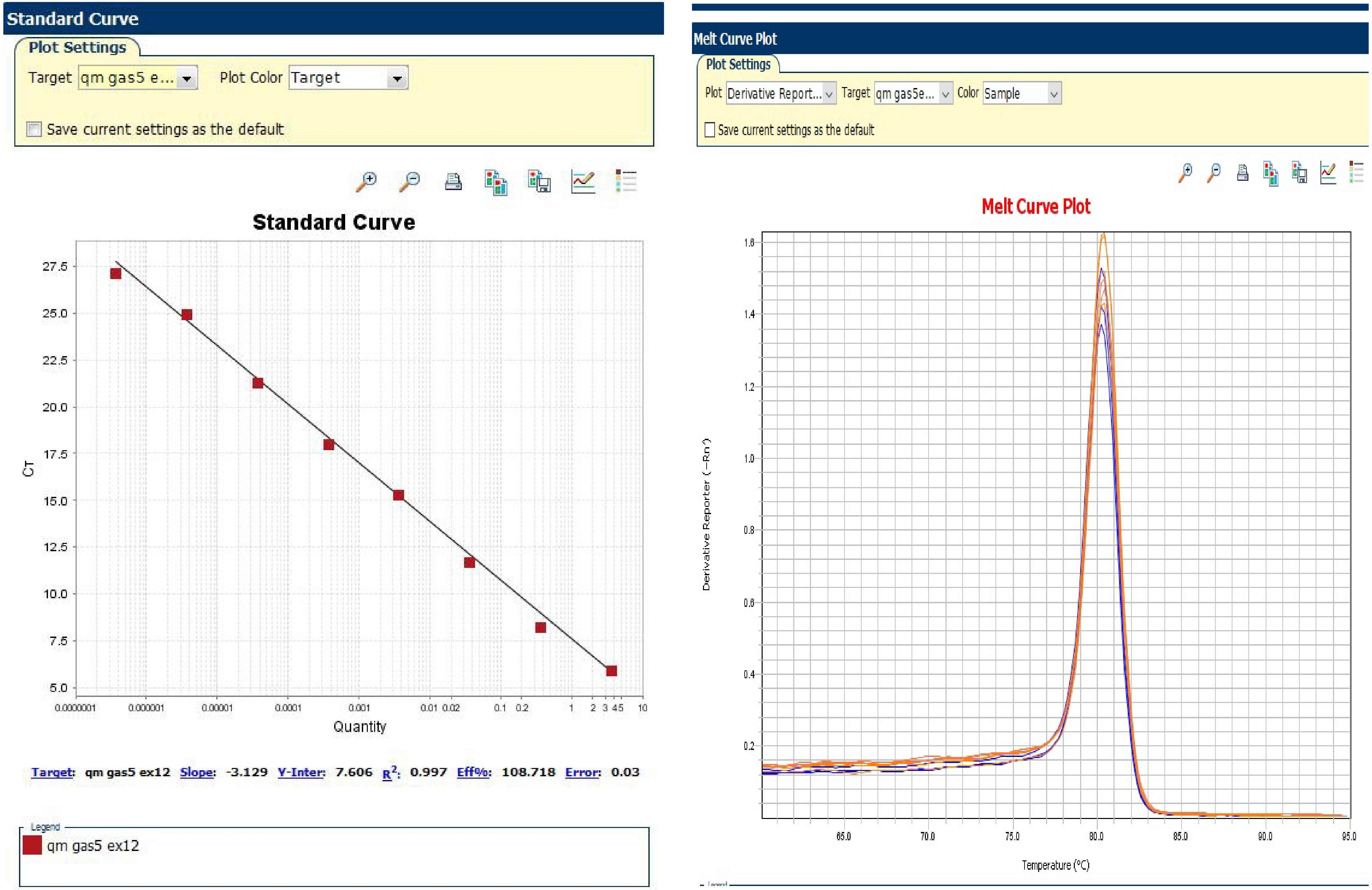

Supplement: Supplementary file 1 [file biology-15-01488-s001.zip › biology-4373375-supplementary.pdf]
